# Diagnostic Efficacy of Sputum Cytology versus Invasive Procedures for Lung Cancer: A Comparative Study

**DOI:** 10.7759/cureus.77829

**Published:** 2025-01-22

**Authors:** Merve Sari Akyuz, Celal Satıcı, Fatma Elif Cayir Kocal, Sinem N Sokucu, Cengiz Ozdemir, Halide Nur Urer

**Affiliations:** 1 Pulmonology, Antalya Training and Research Hospital, Antalya, TUR; 2 Pulmonology, Yedikule Chest Disease and Thoracic Surgery Training and Research Hospital, Istanbul, TUR; 3 Pulmonology, Liv Hospital Vadistanbul, Istanbul, TUR; 4 Pathology, Haseki Training and Research Hospital, Istanbul, TUR

**Keywords:** cytology, diagnosis, lung cancer, lung cancer staging, sputum

## Abstract

Objective and aim: Interventional diagnostic procedures such as bronchoscopy and transthoracic needle aspiration can lead to significant complications, especially in more vulnerable patients. In our study, we aimed to evaluate the characteristics of patients with high diagnostic rates via sputum cytology, a non-invasive technique.

Methods: This retrospective, nested case-control study included patients who were diagnosed with lung cancer and who underwent sputum cytology at least once in our tertiary referral hospital between 2012 and 2022.

Results: There were no significant differences between the groups in terms of age, gender, comorbidities, presence of hemoptysis at admission, smoking history/smoking duration, endobronchial localization, clinical stage, maximum standardized uptake value (SUV_max_), or long tumor diameter (p > 0.05). However, patients diagnosed with sputum cytology were more likely to have adenocarcinoma (81.2% vs. 27.7%, p < 0.001) and less likely to have unclassified non-small cell lung cancer (0% vs. 22.9%, p = 0.03). The mean length of survival for patients diagnosed with sputum cytology was shorter than that for those diagnosed with invasive procedures (34 vs. 160 months, p = 0.03).

Conclusion: Our findings suggest that sputum cytology may have diagnostic efficacy for the adenocarcinoma subtype. Despite the similar clinical stages between the groups, the higher mortality observed in patients diagnosed with lung cancer via sputum cytology may warrant updates in the staging system.

## Introduction

Lung cancer stands as one of the foremost causes of cancer-related mortality [1]. Timely diagnosis and appropriate treatment strategies for patients with lung cancer are paramount. Invasive diagnostic techniques conventional bronchoscopy, rigid bronchoscopy, endobronchial ultrasonography (EBUS) and transthoracic needle aspiration are commonly employed. However, these diagnostic interventions carry the risk of severe complications such as significant hemorrhage, pneumothorax, respiratory failure and even mortality [2,3]. Conversely, sputum cytology, a non-invasive diagnostic approach, presents a viable option for patients suspected of having lung cancer, particularly those with multiple comorbidities, advanced stages of lung cancer or heightened concerns regarding the potential for serious complications.

Sputum remains the only non-invasive method utilized for diagnosing lung diseases, with an optimal sample containing alveolar macrophages [4]. The evidence suggests that the sensitivity of the technique is strongly correlated with the number and adequacy of sputum samples [5]. The evaluation of sputum cytology is typically conducted by cytopathologists. Sputum cytology demonstrates higher diagnostic efficacy, particularly for centrally located tumors larger than 2.4 cm, with the squamous cell subtype, and presenting with hemoptysis upon initial admission [6]. Despite advancements in technology leading to the emergence of novel diagnostic methods, the sensitivity and specificity of conventional cytology combined with molecular analysis in detecting lung cancer have been reported to be 60% and 90%, respectively [7]. Additionally, studies indicate that the integration of sputum cytology with radiological screening or molecular examinations may be advantageous for early disease detection [8].

In certain cases, providing a pathological diagnosis may be more crucial rather than staging in a population where invasive interventional procedures cannot be tolerated and for patients who do not accept invasive diagnostic procedures. Indeed, when the disease lacks histopathological verification, patients may be deprived of the benefits derived from both oncological and palliative treatments. Histopathological confirmation not only guides treatment decisions but also allows for the initiation of appropriate therapeutic interventions tailored to the specific type and stage of the disease. Without such confirmation, the ability to administer targeted therapies, chemotherapy, radiation therapy, or palliative care may be compromised, potentially leading to suboptimal management of the patient's condition and outcomes. However, owing to the limited availability of cytopathologists in many health centers and the associated cost-effectiveness concerns, evaluating sputum cytology as the initial diagnostic step in patients with suspected lung cancer may not be practical. Therefore, it is essential to identify the specific patient populations for whom sputum cytology analysis would be most beneficial.

In line with these findings, we aimed to define the demographic and clinical characteristics of patients who were diagnosed with the use of sputum cytology.

## Materials and methods

Study design and settings

This study was carried out at the Yedikule Chest Diseases and Thoracic Surgery Training and Research Hospital, a tertiary care facility where approximately 5000 patients are diagnosed with lung cancer annually. The study was designed as a retrospective nested case-control study (Ethics Committee approval date: 14.04.2022, Decision no: 2022-212). This article was previously presented as a meeting abstract at the 2022 Solunum Hybrid Meeting on October 10, 2022.

Study population

**Figure 1:**
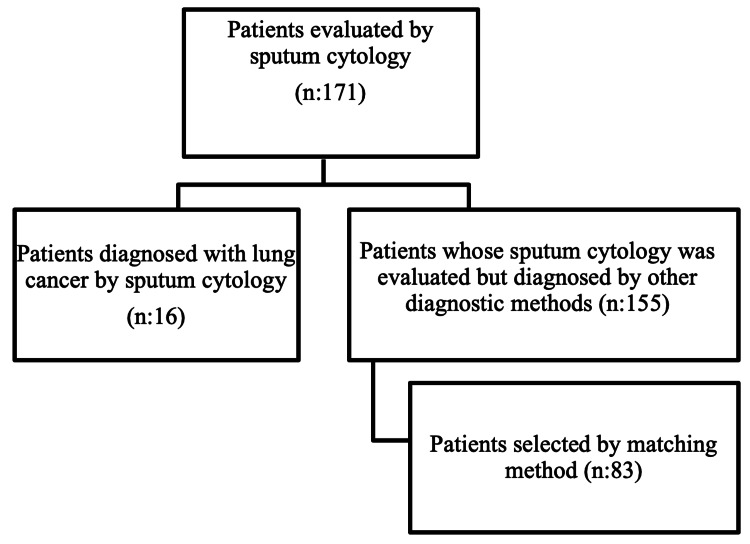
Flowchart of patient selection based on sputum cytology evaluation

The study included patients who were diagnosed with lung cancer in chest diseases clinics between 2012 and 2022, who had undergone sputum cytology at least once, and who were aged between 18 and 85 years. Among these patients, 16 individuals who were diagnosed with lung cancer via sputum cytology were included in the case group. The case group/control group ratio was set at 1:5. Additionally, out of 155 patients who underwent sputum cytology at least once but were diagnosed with lung cancer through other diagnostic methods, 83 were included in the control group through matching. Exclusion criteria encompassed a prior diagnosis of primary lung malignancy or extrapulmonary cancers, incomplete or missing clinical record (Figure 1).

Data collection

Age, sex, smoking history, comorbidities, the presence of hemoptysis at presentation, thoracic CT findings, the location and size of the most proximal tumor in the bronchial tree, positron emission tomography (PET)-CT findings, pathology results, and survival times were assessed through the hospital information system.

Definition

Sputum samples were obtained either spontaneously or via induction. Induced sputum was collected by inhaling 3% NaCl for 15-20 minutes. For cytological examination, the first sputum sample obtained at least twice in the morning is considered ideal [9]. Sputum samples should be processed as soon as possible, preferably within two hours of collection, to ensure accurate cell counting and staining [10]. In the event of a processing delay, the sample may be stored at 4°C for up to eight hours without affecting the number of cells [11]. Typically, the percentage of epithelial cells in induced sputum ranges from 2-11% [12]. Normally, macrophages make up approximately 50% to 80% of the total number of cells [13]. All sputum samples were analyzed by the same cytopathologist.

Patients were classified as having small cell, classified non-small cell carcinoma (adenocarcinoma, squamous cell carcinoma), or unclassified non-small cell carcinoma according to the tumor type. Those in whom suspicious malignant cells were identified on cytological examination but could not be typed were classified as unclassified non-small cell lung cancer [14].

Lung cancer staging was conducted utilizing Tumor, Node, Metastasis (TNM) version 8 criteria, whereas radiological staging was determined based on PET-CT findings. The long diameter of the tumor was measured through multiplanar evaluation [15]. Tumors with ground glass density were measured using the parenchyma window, whereas tumors with solid components were measured via the mediastinum window. Survival was defined as all-cause mortality from the date of pathological confirmation of disease, as documented in patient files.

Our primary aim was to identify the clinical, histological, and radiological characteristics of patients definitively diagnosed through cytological evaluation of sputum. Our secondary aims were determination of the relationship between diagnostic, histological and radiological features and the relationship of all these with survival.

Statistical analysis

A matching technique was employed to create a control group of patients who were diagnosed via invasive techniques. Descriptive statistics are reported as counts and frequencies for categorical variables, means and standard deviations for normally distributed continuous variables, and medians and interquartile ranges for non-normally distributed continuous variables. Categorical variables were compared using chi-square analyses, while continuous variables were compared via Student's t-test for normally distributed data and the Mann-Whitney U test for non-normally distributed data. A p-value < 0.05 was considered significant. Statistical analysis was conducted using SPSS version 20.0. 

## Results

16 patients who were diagnosed with lung cancer through sputum cytology and 83 patients diagnosed through other invasive procedures were included in the study. The mean age of all patients was 62.4 ± 12.0 years, with 10 (10.1%) being female. Most patients diagnosed by sputum cytology were former smokers, while the majority of those in the control group were active smokers. Chronic obstructive pulmonary disease (COPD) was the most common comorbidity in both groups. There were no significant differences between the groups regarding age, sex, comorbidities, presence of hemoptysis at admission, or smoking status/smoking duration (p> 0.05) (Table 1).

**Table 1:** Demographic Characteristics of Patients ^1^ Student's t-test;^ 2^ Chi-square^; 3^ Mann-Whitney U NS: Non-significant; DM: Diabetes mellitus; HT: Hypertension; COPD: Chronic obstructive pulmonary disease, IHD: Ischemic heart disease All statistical results refer to the comparison of patient diagnosed with and without sputum cytology.

Variable	Patients Diagnosed with Sputum Cytology (n: 16)	Patients Diagnosed with Invasive Procedures (n: 83)	P Value
Age (years) (mean ± SD)	66.8 ±14.2	61.6 ±11.4	0.11^1^
Sex			NS^2^
Male, n (%)	15 (93.7)	74 (89.2)	
Female, n (%)	1 (6.3)	9 (10.8)	
Hemoptysis, n (%)	4 (25)	34 (41)	0.26^2^
Smoking Status, n (%)			0.21^2^
Never Smoked	2 (12.5)	11 (13.3)	
Former Smoker	10 (62.5)	33 (39.8)	
Current Smoker	4 (25)	39 (47)	
Smoking (pack/year)	30 (16.2-40)	40 (30-65)	0.058^3^
Comorbidities, n (%)			
DM	2 (12.5)	11 (13.2)	NS^2^
HT	8 (50)	20 (24.1)	0.07^2^
COPD	8 (50)	39 (47)	NS^2^
IHD	4 (25)	15 (18.1)	0.73^2^

In terms of tumor subtypes, patients diagnosed through sputum cytology were more likely to have adenocarcinoma (n: 13, 81.2% vs. n: 23, 27.7%, p < 0.001) and less likely to have unclassified non-small cell lung cancer (n: 0, 0% vs. n: 19, 22.9%, p = 0.03). Most patients in both groups did not exhibit endobronchial involvement of the tumor (n: 9, 56.3% vs. n: 34, 41%). There were no statistically significant differences between the T, N, and M stages of both groups. Among patients diagnosed through sputum cytology, the most common tumor stages were Stages 4B and 3A, whereas in the control group, they were Stages 4B and 4A, respectively. The mean long diameter of the primary tumor was 4.9 cm in the sputum cytology group and 5.3 cm in the control group, with no significant difference observed. Similarly, there was no significant difference between the mean SUV_max_ values of the primary tumor (13.8 vs. 17.2, p = 0.43). The mean survival of patients diagnosed with lung cancer through sputum cytology was shorter than that of patients diagnosed through invasive procedures (34 days vs. 160 days, p = 0.03) (Table 2).

**Table 2:** Clinical and pathological characteristics of patients ^1^ Chi-square; ^2^ Mann-Whitney U NS: Non-significant; SUV_max_: Maximum standardized uptake value.; IQR: Interquartile range

Variable	Patients Diagnosed with Sputum Cytology (n: 16)	Patients Diagnosed with Invasive Procedures (n: 83)	P Value
Lung Cancer Subtype, n (%)			0.001^1^
Adenocarcinoma	13 (81.2)	23 (27.7)	<0.001^1^
Squamous Cell Carcinoma	3 (18.8)	33 (39.8)	0.11^1^
Unclassified Non-small Cell Lung Cancer	0 (0)	19 (22.9)	0.03^1^
Small Cell Lung Cancer	0 (0)	8 (9.6)	0.19^1^
Endobronchial Proximal Localization, n (%)			0.57^1^
No Endobronchial Involvement	9 (56.3)	34 (41)	
Trachea	1 (6.3)	5 (6)	
Main Bronchus	3 (18.8)	20 (24)	
Lobar Bronchus	3 (18.8)	11 (13.3)	
Segmentary Bronchus	0 (0)	13 (15.7)	
Lung Cancer Stage, n (%)			0.38^1^
Stage 1A	0 (0)	5 (6)	
Stage 1B	1 (6.3)	3 (3.6)	
Stage 2A	1 (6.3)	2 (2.4)	
Stage 2B	1 (6.3)	3 (3.6)	
Stage 3A	5 (31.3)	10 (12)	
Stage 3B	2 (12.5)	14 (16.9)	
Stage 3C	0 (0)	8 (9.6)	
Stage 4A	1 (6.3)	16 (19.3)	
Stage 4B	5 (31.3)	22 (26.5)	
T stage, n (%)			0.59^1^
T1	0 (0)	8 (9.6)	
T2	4 (25.7)	22 (26.5)	
T3	1 (6.3)	11 (13.3)	
T4	11 (68.75)	39 (47)	
N stage, n (%)			0.40^1^
N0	6 (37.5)	18 (21.7)	
N1	1 (6.3)	8 (9.6)	
N2	5 (31.3)	20 (24.1)	
N3	4 (25)	36 (43.4)	
M Stage, n (%)			0.93^1^
M0	9 (56.3)	43 (51.8)	
M1a	1 (6.3)	9 (10.8)	
M1b	1 (6.3)	7 (8.4)	
M1c	5 (31.3)	24 (28.9)	
Primary Lung Tumor SUV_max_, Median (IQR)	13.8 (7.4-21.6)	17.2 (9.5-22.5)	0.43^2^
Diameter of the Long Axis of the Tumor (cm), Median (IQR)	4.9 (3-5.3)	5.3 (3.6-7.3)	0.20^2^
Length of Survival Day, Median (IQR)	34 (17.7-243)	160 (35-463)	0.03^2^

## Discussion

Given our limited sample size, the diagnostic role of sputum cytology does not appear to be influenced by factors such as bronchial involvement, clinical stage, and SUV_max_ value. Notably, there is a lack of studies in the literature have evaluated the relationship between the SUV_max_ value of a tumor and the diagnostic efficacy of sputum cytology.

In one study, it was observed that patients with centrally located masses and patients diagnosed with squamous cell carcinoma were diagnosed at a higher rate by sputum cytology [16]. However, Agustine et al. reported that 48% of patients diagnosed via sputum cytology had adenocarcinomas when tumors were not detectable bronchoscopically [17]. Neuman et al. also reported that the number of patients diagnosed with squamous cell carcinoma through cytological evaluation of sputum was higher than that of patients diagnosed with adenocarcinoma, though the difference was not statistically significant [18]. In this study, we may conclude that the diagnostic efficacy of sputum cytology appears to be higher in the adenocarcinoma subtype.

Recently, studies have accelerated that sputum evaluations can be used in the early-stage diagnosis of lung cancer or for screening. In early-stage lung cancer, the detection of cancer cells in sputum and the diagnosis of lung cancer can rarely be performed via conventional cytology alone. However, it has been documented that the assessment of genetic and epigenetic alterations in shed cells may serve as indicators of increased cancer risk [19]. Veena et al. suggested sputum cytology as a non-invasive alternative to biopsy procedures for the differential diagnosis of lung adenocarcinoma [20].

Conventional sputum cytology has been reported to exhibit high sensitivity and specificity in diagnosing lung cancer when combined with molecular analysis. Furthermore, the integration of fluorescence in situ hybridization (FISH) with standard cytology has been shown to improve the sensitivity to 76% and the specificity to 92% for the early diagnosis of lung cancer [7,21].

In the literature, increasing the number of sputum samples analyzed per patient can increase sensitivity. Furthermore, collecting samples over three consecutive days or pooling these samples for analysis has been shown to increase efficiency [22,23].

In our clinic, sputum cytology is utilized primarily in patient groups who do not consent to other interventional methods or who have contraindications for such procedures. However, not all physicians prefer sputum cytology, which is a limitation of this study. In our analysis, 81.3% (n: 13) of patients diagnosed through sputum cytology and 84.3% (n: 70) of those in the comparison group had Stage 3A disease or higher. These findings suggest that sputum cytology can be employed for cytological diagnosis in patients with advanced cancer, as determined by various imaging modalities.

This study is limited by its retrospective design, the small sample size of patients evaluated, and the absence of standardized protocols for sputum sample collection, which may have introduced variability in diagnostic outcomes and affected the generalizability of the findings.

## Conclusions

Sputum cytology remains a valuable diagnostic tool, particularly in patients who are not candidates for invasive procedures. Our findings suggest that sputum cytology may have higher diagnostic efficacy for the adenocarcinoma subtype. Although the groups in our study exhibited similar stages, the elevated mortality rate observed in the patient group diagnosed with malignancy through sputum cytology may prompt considerations for updates to be made in the staging system. Further studies are warranted to optimize its use and enhance diagnostic outcomes in lung cancer detection.
